# Hominin interbreeding and the evolution of human variation

**DOI:** 10.1186/s40709-016-0054-7

**Published:** 2016-07-16

**Authors:** Kwang Hyun Ko

**Affiliations:** Hanyang University, Seoul, South Korea

**Keywords:** Neanderthal, *Homo sapiens*, Denisovan, *Homo heidelbergensis*, Hominin interbreeding

## Abstract

Mitochondrial Eve confirms the “out of Africa” theory, but the evidence also supports interbreeding between *Homo sapiens* and other hominins: Neanderthals, Denisovans, and *Homo heidelbergensis*. This article explains how interbreeding between early *H. sapiens* and archaic hominins occurred. The availability of edible insects in East Asia aided the spread of the unaggressive, highly cooperative Neanderthals, who interbred with *H. sapiens* in Asia, resulting in a higher admixture of Neanderthal DNA in East Asian populations. Geographical variation in degree of interbreeding between *H. sapiens* and Neanderthals likely contributed to neurological and behavioral differences in modern humans. Similarly, people with Denisovan genetic admixture were better able to dwell in mountainous regions, allowing their genetic legacy to cross the Himalayas and persist in Southeast Asian and Oceanian *H. sapiens*. In the Sub-Saharan region, unaffected by Denisovan or Neanderthal interbreeding, *H. sapiens* interbred with *H. heidelbergensis*, because high humidity militated against fire-making and allowed the survival of these non-fire-making hominins.

## Background

There are two branching hypotheses on the origin of the human species. The most widely accepted is the “out of Africa” (OOA) theory, which holds that archaic *Homo sapiens* evolved into anatomically modern humans solely in Africa between 200,000 and 60,000 years ago [1]. This hypothesis further proposes that members of one branch of *H. sapiens* left Africa at some point between 125,000 and 60,000 years ago, and that over a long period, these *H. sapiens* replaced more “primitive” populations of other hominins in Asia or Europe, such as *Homo neanderthalensis* and *Homo erectus* [2].

The competing theory is the multiregional evolution hypothesis [3], which argues that some or all of the genetic variation among contemporary human races is attributable to genetic inheritance from either other *H. sapiens* subspecies or from other hominid species. In the multiregional model, all archaic human forms worldwide, such as *H. erectus* and Neanderthals, as well as modern forms, subsequently evolved together into the diverse populations of modern *H. sapiens*, which are considered to make up a single, continuously gradient (as distinct from categorically separate) human species.

DNA analysis demonstrating the existence of “Mitochondrial Eve” has strongly corroborated the recent African origin model of OOA by providing crucial support to the theory that *H. sapiens* moved from Africa to replace residing hominin populations elsewhere [4]. Mitochondrial Eve is the most recent matrilineal common ancestor of all humans currently alive. Women pass along mitochondrial DNA unchanged during sexual reproduction, and the DNA of this most recent woman from whom all currently living humans descend through an unbroken line on their mother’s side proves that modern humans only evolved once, most likely in East Africa, sometime between 150,000 and 200,000 years ago.

At the same time, however, studies suggest that Neanderthals, our closest-known evolutionary relatives, coexisted with *H. sapiens* on Earth for more than 5000 years and frequently interbred with modern humans [5]. According to researchers, at least one-fifth of the Neanderthal genome may lurk within modern humans, influencing traits including the appearance of the skin and hair people have today and the diseases they get. This finding indicates that a true “extinction” of Neanderthals may not have occurred [6], but that they may have been absorbed into *H. sapiens.* Genetic evidence shows that other archaic hominins, such as the Denisovans, also interbred with *H. sapiens* [7]. The most current version of the OOA hypothesis emphasizes the African origin of most human populations but allows for the possibility of local contributions/interbreeding between humans and other hominins [8]. Consequently, this article mainly discusses Neanderthal–human interbreeding, while also explaining other admixtures of archaic humans with hominins who were their contemporaries, such as Denisovans and *H. heidelbergensis*.

### Origin of race: human interbreeding with Neanderthals and Denisovans

Neanderthals are an extinct species of human (in the genus *Homo*), related to modern humans [9]. Traces left by Neanderthals include bone and stone tools, which have been found all over Eurasia, from Western Europe to central and northern Asia. Neanderthals are generally classified by biologists as *H. neanderthalensis*, and sometimes as *Homo sapiens neanderthalensis.*

Denisovans are another extinct species of humans, similar to Neanderthals. The Denisova Cave is located in southwestern Siberia, in the Altai Mountains near the Russian border with China and Mongolia [10]. Research shows that Denisovans shared a common origin with Neanderthals but were genetically distinct.

Recent genetic studies have shown a higher Neanderthal admixture in East Asians compared with Europeans [11], most likely indicating that at least two independent gene-flow events must have taken place in early modern humans and that the early ancestors of East Asians experienced more admixture than those of Europeans after the divergence of these two groups [12]; to put it in another way, studies seeking to explain why East Asians inherited 15–30 % more Neanderthal DNA than Europeans have concluded that East Asians interbred with Neanderthals in two waves [13].

The first interbreeding with Neanderthals occurred in the Middle East before the ancestors of modern non-Africans spread out across Eurasia. The ancestors of modern Europeans and Asians then split out of this migrant group [12], and the ancestors of East Asians interbred again with Neanderthals after the split. The first humans with proto-Neanderthal traits are believed to have existed in Eurasia as early as 350,000–600,000 years ago, with the first “true Neanderthals” appearing between 200,000 and 250,000 years ago.

As this implies, Neanderthals and Denisovans were likely more closely related to one another than either was to modern humans [14]. Although the range covered by Denisovans is argued, studies have confirmed the impact of Denisovan ancestry in the islands of Oceania, particularly Papua New Guinea, and some parts of mainland Asia, such as Tibet.

### Why does Neanderthal ancestry appear to a higher degree in Asia?

All *H. sapiens* living today have interbred to some degree with Neanderthals, Denisovans, or other hominins, and as outlined above, we know that these hominin groups lasted longer and interbred more in some parts of the world than in other areas.

Most hominins other than Denisovans and Neanderthals were simply replaced by *H. sapiens* that migrated out of Africa, but sufficient interbreeding occurred with Denisovans and Neanderthals in Eurasia to leave a significant mark on modern human DNA [15]. Because Neanderthals ranged only from Europe to West Asia, the question of why there were two waves of interbreeding between East Asian *H. sapiens* and Neanderthals remains a mystery. The answer to this question lies in differences in behavior, and in particular aggressiveness, between groups of hominins.

*Homo heidelbergensis*, which exhibited proto-Neanderthal traits, existed in Eurasia as early as 350,000–600,000 years ago, while the first Neanderthals appeared between 200,000 and 250,000 years ago [16]. At varying times, Neanderthals inhabited the region from Western Europe to Central Asia; their eastern and northern range extended to Okladnikov in the Altai region and Byzovaya in the Ural region of present-day Russia [17]. Neanderthals started to disappear/interbreed with *H. sapiens* from the time the latter migrated to Europe. Fossil findings have indicated brutality and violence among *H. sapiens* living 10,000 years ago [18]. The evidence has shown that in addition to interbreeding, Neanderthals were also very often killed by *H. sapiens*, and in related findings, genetic studies have shown that the mutations in ADSL, GLDC, and SLITRK1 genes, which are associated with hyperactivity and aggressive behavior in modern humans, were not found in Neanderthals [19–21]. Thus, by multiple methods, *H. sapiens* were responsible for the extinction of Neanderthals, who were more cooperative and less aggressive than *H. sapiens* according to studies from various fields.

It should be noted that compared to Africa, Eurasia lacks predators that could have presented a threat to hominin species. The increase in species richness or biodiversity that occurs from the poles to the tropics is often referred to as the latitudinal diversity gradient (LDG), and the greatest biodiversity is found in the tropics [22]. The African continent lies almost entirely within the tropics and extends equally to the north and south of the equator, which creates favorable conditions for wildlife, including large predators [23]. The rich vegetation in Africa, where edible fruits and nuts are abundant, results in a diversity of animal species, and many carnivores, such as hyenas, lions, vultures, crocodiles, and cheetahs, reside exclusively in this biologically diverse region, where they once posed a major threat to human species.

In the Eurasian region, in contrast, where Neanderthals evolved and ultimately ranged, there were no carnivores that regularly preyed on humans. Thus, the Neanderthals that ranged in Eurasia evolved toward peaceful behavior.

Thus, the *H. sapiens* that came after *H. heidelbergensis* began their journey in East Africa, where they had to compete with other animals, including archaic hominins, and watch out for dangerous predators. The hyperactivity and violence of *H. sapiens*, which distinguish them from Neanderthals, were an essential part of their survival, because they had to fight and often kill predators and competitors.

### Edible insects and Asia

Although Neanderthals never inhabited East Asia, East Asians have more Neanderthal DNA than Europeans do. The peaceful nature of Neanderthals would have been advantageous in East Asia due to the large of amount edible insects available—considerably higher than in Europe.

Before a rise in human population density forced people to turn to agriculture, hominins were hunter-gatherers, whose diet mostly consisted of fruits, nuts, and insects. Prior to the domestication of animals in approximately 9000 BCE, hominins would have relied on hunting and scavenging to obtain what meat they ate. Due to the instability of meat supplies obtained from hunting, partly a result of unreliable tools, hominins largely depended on insects for protein; coprolites from caves in the United States and Mexico, containing ants, beetle larvae, lice, ticks, and mites [24], have evidenced this prehistoric entomophagy. Similar to other great ape species that eat insects, then, the evolutionary precursors of *H. sapiens* were also entomophagous, and staple insects represented an important part of their diet.

Prehistoric entomophagy practices have persisted over time. Cave paintings in Altamira, North Spain, dating from approximately 30,000–9000 BCE, depict the collection of edible insects and wild bee nests, seeming to suggest an entomophagous society. Cocoons of wild silkworm (*Theophila religiosae*) found in ruins in the Shanxi Province of China dateback to 2000–2500 years BCE. The cocoons were discovered with large holes, suggesting that the pupae had been eaten. The eating of insects is still observed in the developed nations of modern-day Asia [25].

**Fig. 1:**
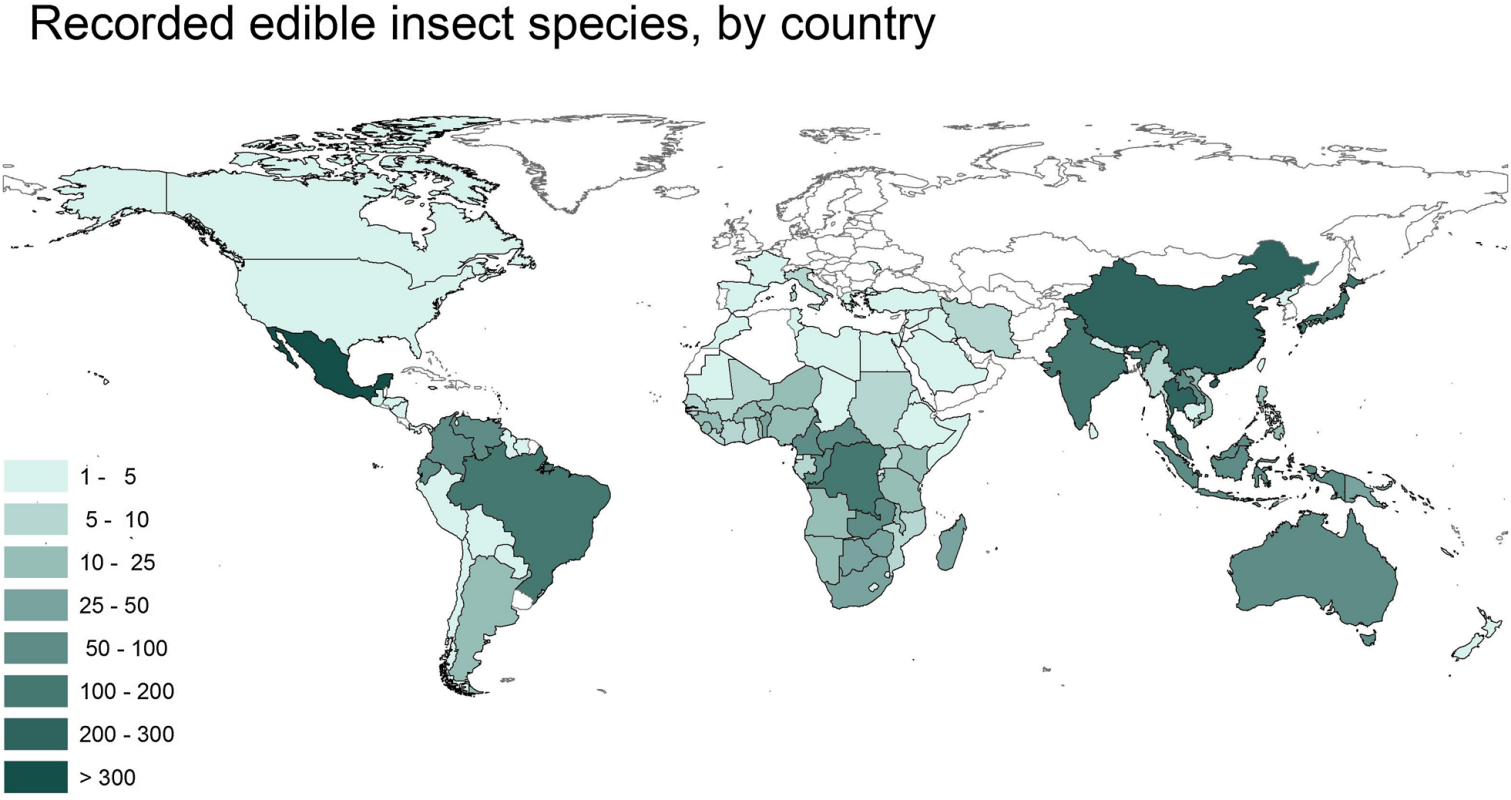
Number of edible insect species by country [26]. Source: Centre for geo information, Wageningen University, based on data compiled by Jongema 2012

The ancestors of East Asians interbred with Neanderthals a second time after the earlier interbreeding in the Middle East, as mentioned above. Compared with Europe, the insect-abundant regions of East Asia were able to support larger populations of hominins. In such an environment, aggressiveness and violence would have been more disadvantageous for survival (Fig. 1).

### Denisovan interbreeding

Denisovans, which were related more closely to Neanderthals than to humans, also interbred with *H. sapiens*. Genetic variation of Denisovans is low compared to that of *H. sapiens*, but Denisovans were present in large parts of Asia for possibly more than 110,000 years, allowing *H. sapiens* in Asia to obtain Denisovan traits from interbreeding [7].

Evidence indicates that the highest Denisovan admixture is found in Oceanian populations, followed by many Southeast Asian populations, but recent research has also found indications that parts of mainland Asia, such as Tibet, have small traces of Denisovan DNA [27].

Denisovans were adapted to surviving at high altitudes, and Denisovan fossils have been found in high caves in Siberia; researchers have further discovered that Tibetans are inheritors of the ancient Denisovan trait of being able to regulate blood oxygenation [28]. The highest levels of interbreeding with Neanderthals, which were genetically closer to Denisovans than *H. sapiens* were, occurred in East Asia, and East Asians show a small, relatively insubstantial fraction of Denisovan ancestry. Significant levels of Denisovan genes, however, remain in non-East Asian populations (Southeast Asians and Melanesians) residing far from the Denisova Cave in Siberia.

Interbreeding with Denisovans significantly affected *H. sapiens* populations on the island of New Guinea, where the highest mountains and highlands in Australasia are found. In general, populations that proliferated in these mountain ranges would have also spread to nearby regions, leaving remnants of Denisovans throughout Asia. At the end of the Ice Age, the separation of the Sahul and Sunda shelves from mainland Asia, caused by rising sea levels, resulted in the local population of Oceania (and parts of Southeast Asia) being less affected by the admixture of Neanderthals with *H. sapiens*. Thus, traces of Denisovans were more perceptibly preserved in these regions.

### Why Neanderthals lost out to *Homo sapiens*

Although Neanderthals did interbreed with *H. sapiens*, the majority of their population went extinct from competition with *H. sapiens*. As follows from the possession by *H. sapiens* of a mutated gene related to aggression, fossil evidence reveals that Neanderthals were killed by *H. sapiens* in acts of violence [20]. Furthermore, although Neanderthals possessed brain development enabling greater visual acuity than *H. sapiens*, the latter had better language-processing abilities [29]. In general, because Neanderthal brains were devoted to vision and spatial memory, this left less area for cognition and social interactions.

## Interbreeding and race

People from East Asian countries have approximately 20 % more Neanderthal DNA than Western Europeans, and these differences in levels of interbreeding with Neanderthals caused certain neurological differences observed today [16].

A recent study conducted by Park & Huang [30] showed evidence of cultural differences between Westerners and East Asians, resting in differences in areas deep in the brain. Biologically, White American adults showed increased activation in areas related to language and reasoning, such as Wernicke’s and Broca’s areas, whereas East Asians presented stronger activity in perceptual regions, such as the visual-premotor association area [31]. Similarly, European brains have to work harder at relative judgment, whereas East Asian brains find absolute judgments more challenging [32]. In addition, adults from Western cultures process information analytically by focusing on key features, whereas adults from the East process information in a more holistic manner [33]. One of the psychologists who conducted that study states that Westerners look at the focal object more rapidly and spend more time looking at it, whereas Chinese individuals have more saccades, which means that they move their eyes more, particularly back and forth between the object and the background [33].

Furthermore, Neanderthals were less aggressive and more “autistic” than *H. sapiens*. Genes related to hyperactivity and aggression are, in fact, only found in *H. sapiens*. Asians and Pacific Islanders present less symptoms linked to hyperactivity and aggressiveness, such as attention deficit hyperactivity disorder (ADHD), and East Asians score the lowest in terms of aggressive behavior. In the United States, both immigrants of East Asian origins and mainland Asians show lower crime rates compared with Black and White populations [34].

### The significance of *Homo heidelbergensis*

Neanderthals, Denisovans, and modern humans (*H. sapiens*) are all descended from *H. heidelbergensis*. Between 300,000 and 400,000 years ago, one branch of this group became independent of other hominins; some of this group left Africa [35]. One (sub)group branched northwest into Europe and West Asia and eventually evolved into the Neanderthals, while the other group ventured eastward throughout Asia, eventually developing into the Denisovans. The remaining members of this group, *H. heidelbergensis*, evolved into *H. sapiens* approximately 130,000 years ago in the dry savannahs in Africa, and then themselves migrated to other regions and continents [36]. These humans were more adept at controlling fire than the preceding African hominins had been, but the humid tropical regions did not foster the development of fire-making. *Homo sapiens* that settled in the tropics of South Asia and Africa were genetically influenced by the abilities of the anteceding hominins in those regions, who were less dependent on fire-making.

### A short “prehistory”—before *Homo heidelbergensis*

Before *H. heidelbergensis* appeared, *H. erectus* originated in Africa and spread throughout Eurasia, as far as present-day Georgia, India, Sri Lanka, China, and Java. The *H. erectus* who remained in Africa is now widely accepted as the direct ancestor of all later hominins, including *H. heidelbergensis*, *H. sapiens*, *H. neanderthalensis*, and the Asian *H. erectus* [16]. The group that eventually became *H. heidelbergensis* in Africa had established populations in Europe and South Asia by approximately 500,000 years ago.

By approximately 300,000 years ago, regional differences began to develop as these *H. heidelbergensis* adapted to their new environments, having collectively become independent of other hominins shortly after leaving Africa. At this point, one group became the Neanderthals, and another group developed into the Denisovans. The *H. heidelbergensis* remaining in Africa evolved into *H. sapiens* [37].

*Homo sapiens* eventually spread from Africa into Eurasia and replaced the residing hominins; however, a considerable degree of interbreeding with archaic hominins also occurred. Long before the appearance in Eurasia of *H. heidelbergensis* and the Neanderthals, the Denisovans, and ultimately *H. sapiens,* the Asian *H. erectus* inhabited an overlapping area [38], until it was replaced by those successor species and others. However, *H. erectus* on the mainland went extinct long before the arrival of *H. sapiens*, and so the influence of any admixture of *H. erectus* with *H. sapiens* via Neanderthals and Denisovans would be negligible.

### Africa and history of interbreeding

*Homo sapiens* interbreeding with Neanderthals or Denisovans did not occur in the Sub-Saharan regions, although the Khoisan and Yoruba peoples were influenced by a Neanderthal-influenced Eurasian heritage [39]. Neanderthals and Denisovans never lived in Sub-Sahara and never left a genetic mark on Sub-Saharan regions, but interbreeding between Sub-Saharan Africans and an as-yet-unknown hominin, such as *H. heidelbergensis*, has been suggested.

## Fire-making and humidity

The use of fire marked a turning point in human evolution. The fire drove away predators and insects and provided additional warmth to humans. Importantly, cooking with fire allowed humans to conserve energy during digestion, because less energy is spent digesting or chewing cooked foods. One study [40] states that the energetic benefit of consuming cooked foods is very high; further, another study [41] found that mice given cooked meat gained 29 % more weight than mice fed raw meat over a period of 5 weeks, even though the latter consumed more meat. Furthermore, with the use of fire, formerly indigestible or toxic components of plants, such as mature roots, tubers, raw cellulose, thick stems, large leaves, and seeds, became part of the hominid diet; and hominins saved energy not only on digesting but also on foraging and chewing [40]. Hominid brain size increased steadily overtime, but starting at least a million years ago, the rate of increase sped up, a change explained by the early control of fire exhibited by *H. erectus* and the subsequent nutritive benefits. The current archaeological evidence shows at least million years of widespread fire-making, but the actual beginning of fire-making would have been much earlier.

### Conditions for fire-making

Fire provides extra calories by making foods easier to digest, but fire-making is a difficult task that the earliest hominins could not accomplish. One important condition for fire-making is low humidity: if relative humidity (RH) is high, it is hard for moisture to evaporate [42], and at high humidity, fuels will absorb more moisture, making ignition more difficult. Wild fires occur more frequently in regions with low humidity, because fuels become drier. According to the Köppen–Geiger climate classification system, some parts of Africa, South America, and Oceania are classified as tropical humid, more specifically, tropical rainforest, tropical savannah, or tropical monsoon. This classification takes into account annual and monthly temperatures, precipitation, and seasonality of precipitation [43]. In general, the most humid places on Earth are located in the tropics, due to their proximity to the equator (leading to more sun and warmth) and high precipitation levels. In these regions, fire-making is difficult.

**Fig. 2:**
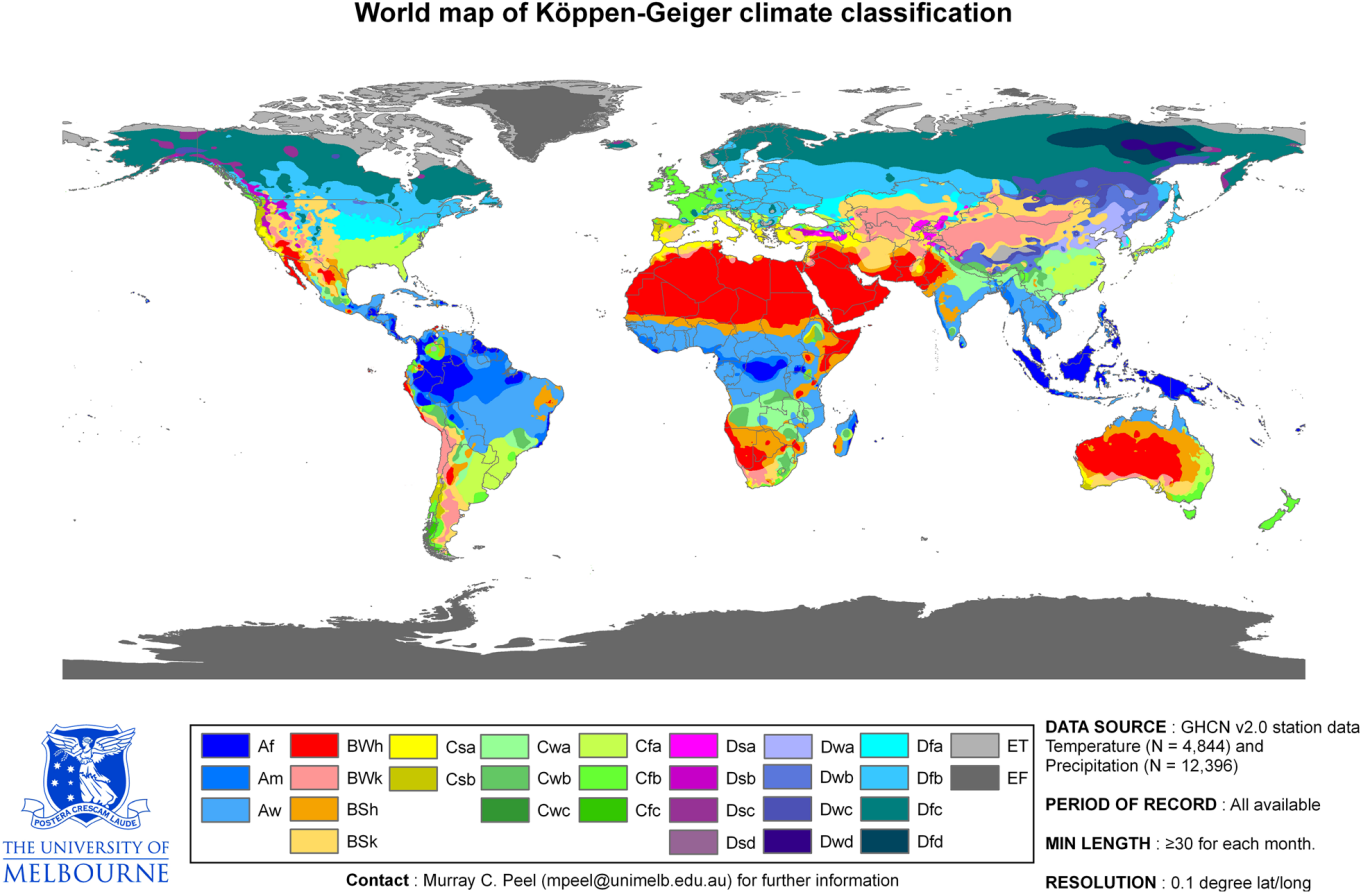
Köppen climate classification. Tropical/megathermal climates, Af/Am/Aw, feature high rainfall, hot temperatures [45]

A correlation can be detected between climate zone and brain size: native populations in tropic zones have smaller brains than people from other zones [44]. The link between brain size and intelligence is, of course, a controversial issue, and larger brains do not necessarily correlate with greater intelligence. However, the development of intelligence derived from fire-making and better nutrition would certainly have been hampered in tropical areas, where the relative humidity is very high. Thus, the survival of hominin species in tropical regions would have been more difficult given the lack of recourse to fire and resulting benefits, and these people would have depended more on cognitive capacity developed from foraging and watching out for predators (Fig. 2).

### Brain size and interbreeding

*Homo heidelbergensis* mostly had smaller brains than Neanderthals, Denisovans, and *H. sapiens*. While no traces of *H. sapiens* interbreeding with Neanderthals or Denisovans have been detected in Sub-Saharan Africa, as noted, genetic studies show that *H. sapiens* did interbreed with *H. heidelbergensi*s in this region [46]. Brain size differences have been noted among people of Sub-Saharan African descent, Australian Aboriginals, and people of European descent, the former two of which showed smaller brains [44]. There are, however, exceptions in Sub-Saharan Africa. Researchers have found traces of western Eurasian Neanderthal-associated DNA in the southern African Khoisan and Yoruba peoples, who would then have migrated back into Africa from the Middle East after the introduction of a Eurasian heritage [39].

The smaller brain sizes detected in aboriginal populations in the tropical regions of Africa and South Asia can be attributed to interbreeding with *H. heidelbergensis* [46]. These humans presumably benefited from interbreeding with hominin ancestors who depended less on fire and likely had cognitive aptitudes making them more adept at surviving in the tropics.

**Fig. 3:**
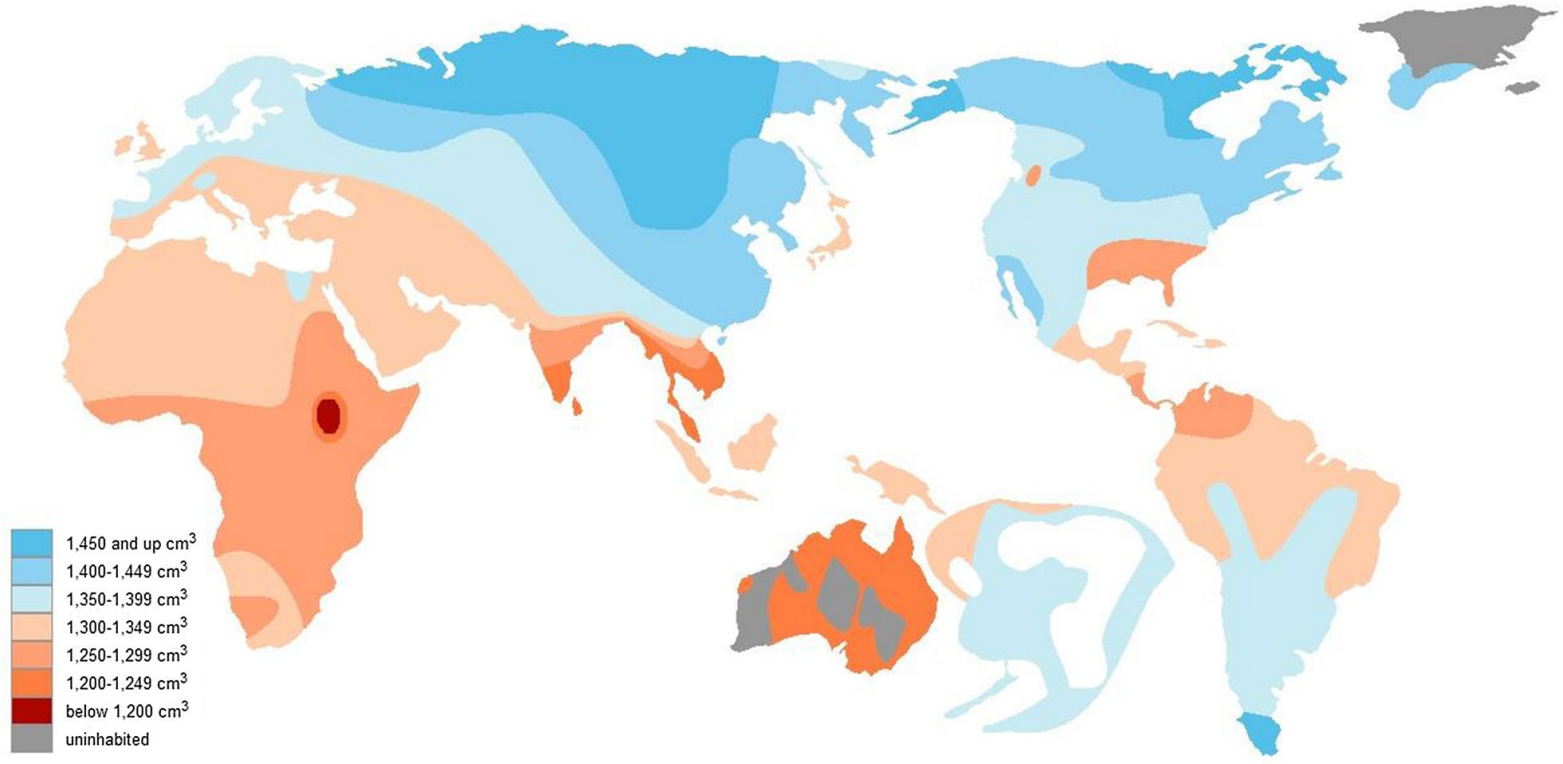
Connection between average cranial capacity and geographic ancestry of *H. sapiens* [44]

A correlation between IQ and brain size has been observed in some studies, but this controversial finding is far from conclusive [47]. The definition of intelligence itself is arguable and IQ as a measure has come under various kinds of criticism; the organization of the individual brain may matter more than the brain size. Regardless, and for what it is worth, studies of brain size based on cranial capacity show lower values in the tropical regions of Africa, South Asia, and Oceania, regions where fire-making would have been difficult, as this section has discussed (Fig. 3).

Furthermore, without an archaic Neanderthal ancestry, Sub-Saharan African populations would be more affected by genes linked to aggression or hyperactivity, which are found only in *H. sapiens*. This finding could help account for the differences in violence or aggression found in the human race today.

Even so, any implications would not be significant. Many cross-cultural exchanges have occurred in the last few 100 years. The geneticist David Reich states that he is unsure whether there is any population that does not have any Eurasian DNA [39]. The study will only provide information regarding Neanderthal DNA acquired thousands of years ago.

## Interbreeding and the human race: conclusion

East Asians have slightly larger brains than Europeans [48]. In more detail, the perceptual brain regions are larger in East Asians, whereas the regions related to language are larger in Europeans. Sub-Saharan Africans mostly have smaller brains than the aforementioned two groups. The significant differences in brain size observed between tropical and other regions were influenced by tropical conditions, which prove harsh for fire-making, a key technology for accessing the nutrition necessary to grow big brains. Instead, *H. sapiens* in tropical regions interbred with populations that depended less on fire for survival, leading to smaller brains in these areas.

In terms of hyperactivity and aggression, the lowest scores among present human populations are obtained by East Asians, followed by Europeans and then Sub-Saharan Africans [49]. The genes pertaining to aggressiveness and hyperactivity originate with *H. sapiens*, and interbreeding with Neanderthals led to more peaceful behavior in humans inheriting Neanderthal genes. Previous studies have indicated that Neanderthal interbreeding did not affect Sub-Saharan populations, with the exception of a few tribes that migrated back from the Middle East.

To sum up, traces of archaic hominin ancestry have been detected in local populations, but the implications of interbreeding for the modern population should not be used to justify racial stereotypes. In multicultural communities, races have mixed to the point that sources of past interbreeding among hominin groups have become insignificant. In addition to the countless historical migrations that have occurred, *H. sapiens* themselves have continued to evolve separately in different regions, and they are not the same humans that lived 10,000 years ago. Nonetheless, many local, isolated aboriginal populations in Africa, Eurasia, and Oceania have remained relatively stable, and their brain sizes show traces of past interbreeding [48].

### Addendum to the conclusion: *Homo floresiensis*

In addition to *H. heidelbergensis*, Neanderthals, and Denisovans, there is another species of archaic hominin that should be considered, one with a very small brain that lived up until recently (around 10,000 years ago). *Homo floresiensis* was a distinct ancient species of hominin discovered on Flores, an island in Indonesia. The most important identifying features of *H. floresiensis* are its “hobbit-like” features, namely its 3.5-foot-tall (1.1-m-tall) body and 380-cm^3^ cranial capacity, as determined from a LB1 specimen [50]. Recent evidence indicates that these hobbit-like specimens evolved from a *H. erectus*-like hominin; Brown [51] suggested that the limited food environment in Flores favored “insular dwarfism,” which resulted in *H. erectus* evolving into a smaller body size. However, why and how this archaic hominin lasted longer than the others remains unclear.

The answers lie in the geographical location of Flores. Flores, which is one of the Wallacean Islands, lies east of the Wallace Line, a faunal boundary line separating it from the Sundaic region to the west, which was exposed to the air during the last Ice Age, from approximately 110,000–12,000 years ago [52]. The presence of fossils of stegdons (elephant-like mammals) on Flores has led experts to hypothesize that the island was formerly linked to the mainland by a short-lived land bridge or stretches of small sea that were crossable by primitive rafts [53]. However, when the Ice Age ended, the rising ocean levels submerged much of the Sundaic region and Wallacean Islands [54]. The resulting longstanding separation from the surrounding continents has severely limited the ability of small animal species to disperse either into or away from the islands.

Before *H. sapiens* was able to construct effective boats to cross large bodies of water such as oceans, a group forming an archaic hominin population that eventually became *H. floresiensis* was isolated from the Asian mainland [55]. As waves of *H. heidelbergensis* and eventually *H. sapiens* from Africa replaced and interbred with local populations in Asia, this isolated group on the small island of Flores remained unaffected by the rapid evolution fueled by hominin ancestors from Africa that could use various tools and fire. Until *H. sapiens* created efficient rafts to cross the oceans and eventually drove away and killed the regional population of *H. floresiensis*, this species of hominin, similar to early hominins, remained in Flores.

## Abbreviations

ADHD: attention deficit hyperactivity disorder
LDG: latitudinal diversity gradient
OOA: out of Africa
RH: relative humidity
